# The N-terminal region of RTP1S plays important roles in dimer formation and odorant receptor-trafficking

**DOI:** 10.1074/jbc.RA118.007110

**Published:** 2019-08-08

**Authors:** Yosuke Fukutani, Ryohei Tamaki, Ryosuke Inoue, Tomoyo Koshizawa, Shuto Sakashita, Kentaro Ikegami, Ikuroh Ohsawa, Hiroaki Matsunami, Masafumi Yohda

**Affiliations:** ‡ Department of Biotechnology and Life Science, Tokyo University of Agriculture and Technology, Koganei, Tokyo 184-8588, Japan; § Department of Molecular Genetics and Microbiology, Duke University Medical Center, Durham, North Carolina 27710; ¶ Biological Process of Aging, Tokyo Metropolitan Institute of Gerontology, Tokyo 173-0015, Japan; ‖ Department of Neurobiology, Duke Institute for Brain Sciences, Duke University Medical Center, Durham, North Carolina 27705; ** Institute of Global Innovation Research, Tokyo University of Agriculture and Technology, Koganei, Tokyo 184-8588, Japan

**Keywords:** molecular chaperone, dimerization, G-protein–coupled receptor (GPCR), membrane transport, flow cytometry, odorant receptors, receptor-transporting protein

## Abstract

Receptor-transporting protein 1S (RTP1S) is an accessory protein that mediates the transport of mammalian odorant receptors (ORs) into the plasma membrane. Although most ORs fail to localize to the cell surface when expressed alone in nonolfactory cells, functional expression of ORs is achieved with the coexpression of RTP1S. However, the mechanism for RTP1S-mediated OR trafficking remains unclear. In this study, we attempted to reveal the mode of action and critical residues of RTP1S in OR trafficking. Experiments using N-terminal truncation and Ala substitution mutants of RTP1S demonstrated that four N-terminal amino acids have essential roles in OR trafficking. Additionally, using recombinant proteins and split luciferase assays in mammalian cells, we provided evidence for the dimer formation of RTP1S. Furthermore, we determined that the 2nd Cys residue is required for the efficient dimerization of RTP1S. Altogether, these findings provide insights into the mechanism for plasma membrane transport of ORs by RTP1S.

## Introduction

Olfaction is mediated by olfactory receptors (ORs)^4^ expressed on the cilia membrane of olfactory sensory neurons (OSNs) in the olfactory epithelium (1, 2). ORs bind odor molecules in the environment and transmit the information directly to glomeruli in the olfactory bulb before reaching higher brain areas where odor perception is constructed (3). ORs constitute the largest family of G-protein–coupled receptors (GPCRs). Humans have ∼400 OR genes, whereas mice have more than 1100 (4, 5). Irrespective of the existence of many OR genes, each mature OSN expresses only one OR (one cell–one receptor rule) (6). The selection and functional expression of a single OR are essential for the maturation of OSN (7).

Many ORs fail to be transported into the plasma membrane and remain in the endoplasmic reticulum when they are expressed heterologously in nonolfactory cells (8, 9). Receptor-transporting protein 1S (RTP1S), the short form of RTP1, and RTP2 were found to be chaperones that mediate the traffic of ORs into the plasma membrane of OSN. RTPs play essential roles not only in OR expression but also in the OR gene choice (10–12). When expressed heterologously in mammalian cells or yeasts, RTPs facilitate the transport and functional expression of many ORs to the plasma membrane (13). This discovery enabled researchers to perform functional characterization of ORs, including high-throughput ligand screening (14–18). RTP1S and RTP2 are abundantly expressed in olfactory tissues and may also be present in other tissues where functional ORs are ectopically expressed. The other two RTP members, RTP3 and RTP4, are expressed in nonolfactory organs where they are involved in the expression of other GPCRs (19, 20). There are no obvious RTP homologs outside the vertebrate species (10).

The amino acid sequences of RTP family proteins are highly conserved. The RTP family consists of type II transmembrane proteins with a cytoplasmic N terminus and a transmembrane domain (TM domain) close to the C terminus. The TM domain and the outer cellular region of RTP1S seem to play a role as the signal promoting OR localization in a lipid raft on the cell membrane (21). Although the TM domain was thought to be essential for the complete function of RTP1S, TM domain deletion mutants (RTP1S_C3 (1–198 amino acids) and RTP1S_C2 (1–176 amino acids)) partially retained the ability to promote OR localization in the plasma membrane (21). Immunoprecipitation analysis indicated that RTP1S forms complexes with ORs (21). However, the interaction mechanism between ORs and RTP1S is unclear partly due to the lack of structural information of the RTP family proteins as well as ORs. To further analyze the maturation mechanism of ORs, the structural information of RTP1S is indispensable.

In this study, we examined the role of the N-terminal amino acids in OR traffic activity. We also performed expression, purification, and structural characterization of the intracellular domain of RTP1S. The recombinant intracellular domain of RTP1S formed a dimer. We also provided evidence for the dimer formation of RTP1S in mammalian cells and demonstrated that the 2nd Cys residue is required for the efficient dimerization of RTP1S.

## Results

### Identification of specific residues that affect the function of RTP1S

**Figure 1.**
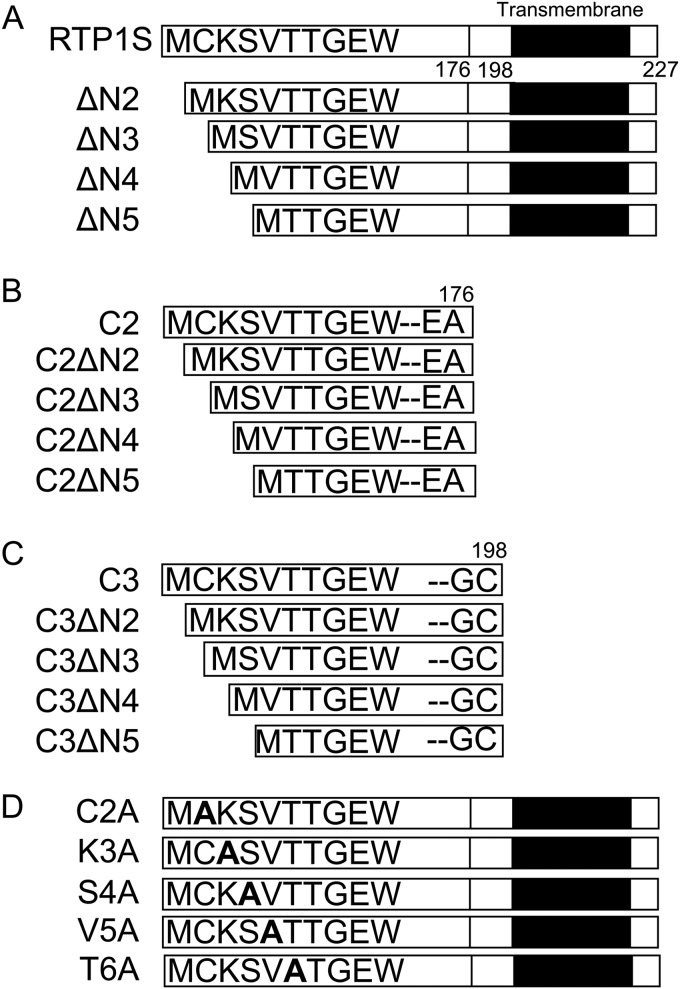
**Diagram of the RTP1S variants used in this study.** N-terminal truncation of RTP1S (*A*), RTP1S_C2 (*B*), and RTP1S_C3 (*C*) is shown. The N-terminal Ala replacement mutants of RTP1S is shown (*D*). The TM domain is shown as a *black bar*.

A previous study showed that the truncation of nine N-terminal amino acids significantly impaired the OR traffic activity of RTP1S (21). To identify the residues in the N-terminal region responsible for OR trafficking, we created a series of N-terminal truncation mutants up to the 6th residue of RTP1S (Fig. 1) and examined their abilities for trafficking ORs. Because ORs exhibit different RTP1S dependence, we used three different ORs: Olfr599, which was used in previous reports (21); Olfr1377; and Olfr1484 (22).

**Figure 2.**
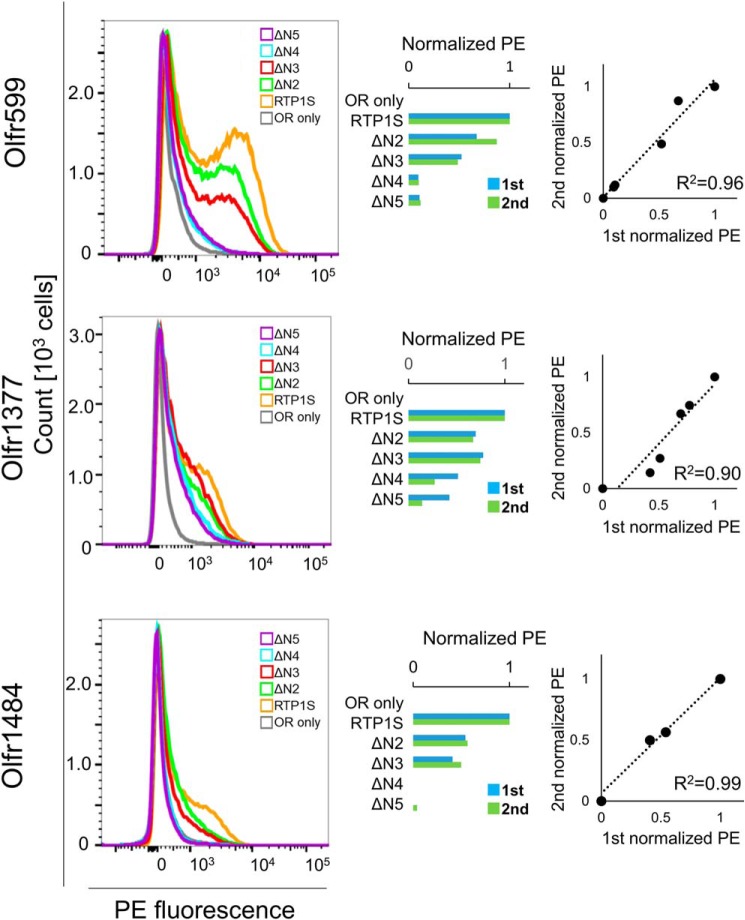
**Cell-surface expression of the Rho-tagged ORs cotransfected with the RTP1S N-terminal truncation mutants.** Each combination of mutant RTP1s and ORs (Olfr599, Olfr1377, and Olfr1484) was transfected into HEK293T cells, and the cell-surface expression level was measured. The results from two independent experiments are shown in each *graph. x* axis, PE fluorescence; *y* axis, cell number. The geometric mean of PE fluorescence of each measurement was used for comparison. The values of RTP1S and OR only were used as positive (= 1) and negative controls (= 0), respectively. Comparison of the normalized response was by different experiments. *R*^2^ values were calculated by Pearson's correlation coefficient from the linear regression analysis.

**Figure 3.**
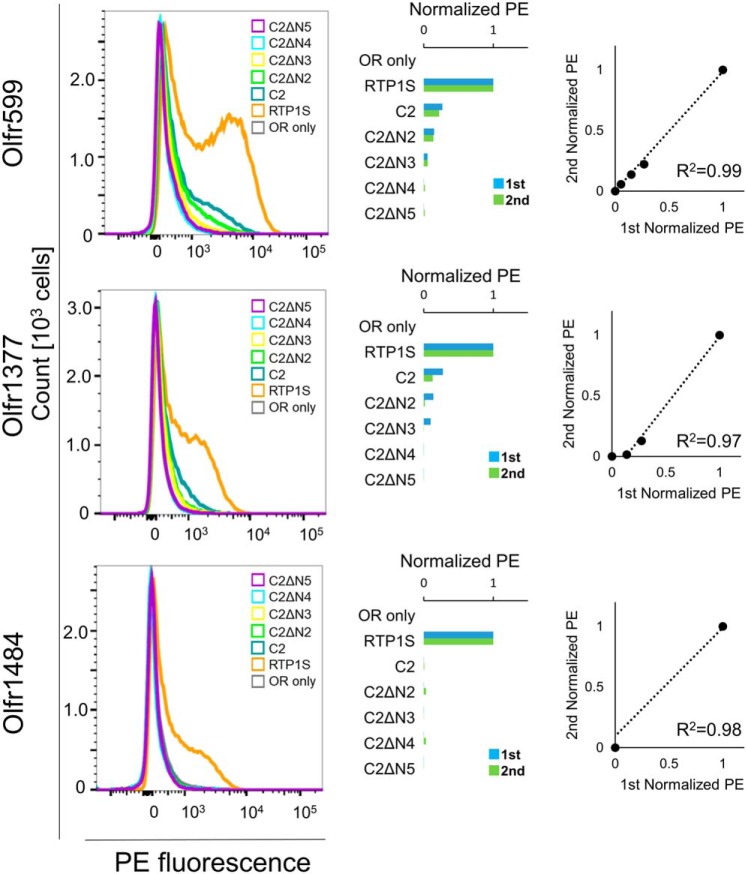
**Cell-surface expression of the Rho-tagged ORs cotransfected with the N-terminal truncation mutants of RTP1S_C2.** Each combination of mutant of RTP1_C2s and ORs (Olfr599, Olfr1377, and Olfr1484) was transfected into HEK293T cells, and the cell-surface expression level was measured. The results from two independent experiments are shown in each *graph. x* axis, PE fluorescence; *y* axis, cell number. The geometric mean of PE fluorescence of each measurement was used for comparison. The values of RTP1S and OR only were used as positive (= 1) and negative controls (= 0), respectively. Comparison of the normalized response was by different experiments. *R*^2^ values were calculated by Pearson's correlation coefficient from the linear regression analysis.

Cell-surface expression of ORs with the coexpression of RTP1S truncation mutants was analyzed by FACS analysis. Truncation up to Ser-4 (RTP1SΔN4) weakened the OR trafficking activity of RTP1S (Figs. 1*A* and 2). The effect increased with the number of truncations. Cell-surface expression of Olfr1484 was undetectable when the RTP1SΔN4 mutant was coexpressed. Similar results were observed for Olfr1377 and Olfr599. However, they exhibited detectable cell-surface expression with RTP1SΔN4. We next tested the effect of N-terminal truncation on the TM domain deletion mutant, RTP1S_C2, to further clarify the functional domains of RTP1S (Figs. 1*B* and 3). Interestingly, RTP1S_C2 was unable to transport Olfr1484 into the plasma membrane, although it retained a weak ability to traffic Olfr599 and Olfr1377. Similar to the RTP1S N-terminal truncation mutants, the truncation of four amino acids abolished the OR trafficking ability of RTP1S_C2. Another TM domain deletion mutant, RTP1S_C3, with a more extended cytoplasmic domain exhibited almost the same results as RTP1S_C2 (Fig. 1*C* and Fig. S1). Thus, the 21 amino acids from the 177th to the 198th amino acid of RTP1S before the TM domain seem to have no significant role in OR transportation into the plasma membrane.

**Figure 4.**
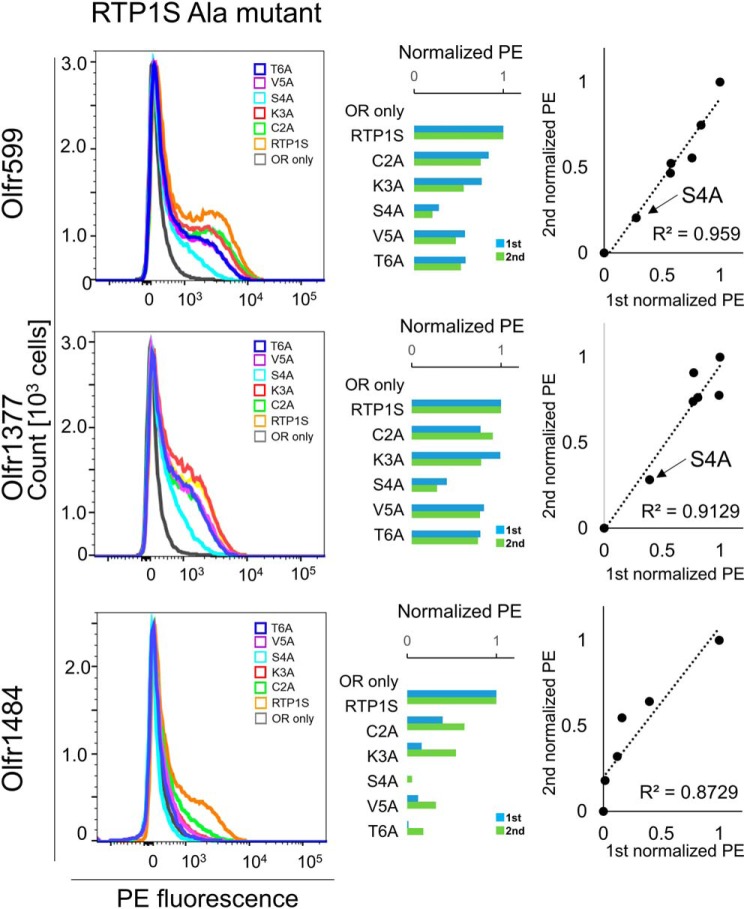
**Cell-surface expression of the Rho-tagged ORs cotransfected with the N-terminal Ala replacement mutants of RTP1S.** Each combination of mutants of RTP1s and ORs (Olfr599, Olfr1377, and Olfr1484) was transfected into HEK293T cells, and the cell-surface expression level was measured. The results from two independent experiments are shown in each *graph. x* axis, PE fluorescence; *y* axis, cell number. The geometric mean of the PE fluorescence of each measurement was used for comparison. The values of RTP1S and OR only were used as positive (= 1) and negative controls (= 0), respectively. Comparison of the normalized response was by different experiments. *R*^2^ values were calculated by Pearson's correlation coefficient from the linear regression analysis.

**Figure 5.**
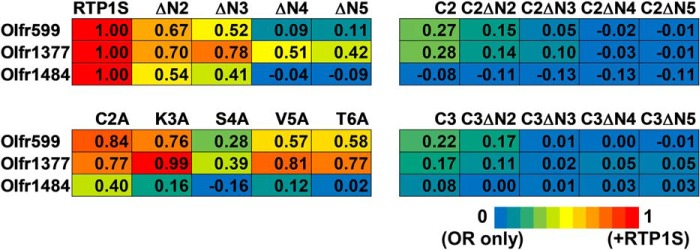
**Heat map of the normalized geometric mean of PE fluorescence of the Rho-tagged ORs cotransfected with each RTP1S mutant.** The values of RTP1S and OR only were used as the positive control (= 1; *red*) and the negative control (= 0; *blue*), respectively.

**Figure 6.**
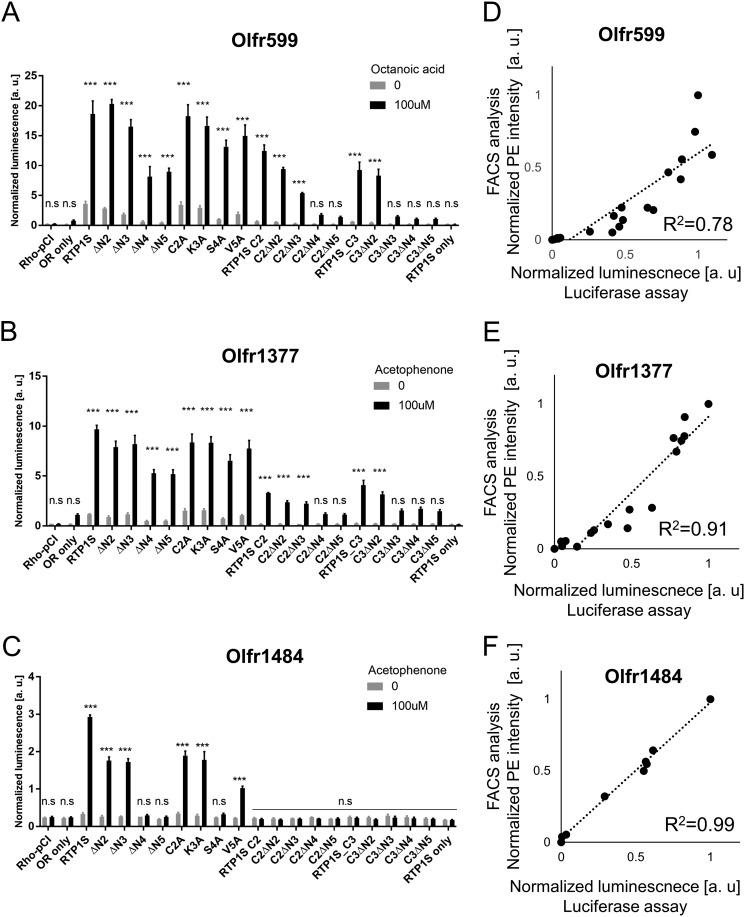
**Correlation between surface expression and ligand response.** Olfr599 (*A*), Olfr1377 (*B*), and Olfr1484 (*C*) were functionally coexpressed with the RTP1S mutants, as indicated by cAMP-mediated luciferase assays. A concentration of 100 μm odorants (caprylic acid for Olfr599 and acetophenone for Olfr1377 and Olfr1484) was used to stimulate ORs. Comparison of the analysis of the response against 100 mm of each odorant in the Luciferase assay and the cell surface expression level in the FACS analysis about Olfr599(D), Olfr1484(E) and Olfr1377(F). The straight line (black dot) is regression line and R2 indicates Pearson's correlation coefficient.

To further evaluate the role of each amino acid residue at the N terminus of RTP1S in OR trafficking, we created Ala replacement mutants and coexpressed them with three different ORs (Figs. 1*D* and 4). Among them, the effect of the S4A mutation was the most significant (Fig. 5). Concerning the responsiveness of the ORs, the surface expression efficiencies of three ORs with RTP1S variants were plotted against their ligand responses (Fig. 6, *A–C*). Olfr1484 was unable to respond against acetophenone in the presence of RTP1S_S4A. We observed linear correlations with significantly high coefficients (*R*^2^ = 0.78–0.99) (Fig. 6, *D–F*).

We also checked the protein expression level of all N-terminal truncation or Ala replacement mutants. Essentially, the expression of all mutants was not affected (Fig. S2). RTP1SΔN2, RTP1SΔN4, RTP1S_C2A, and RTP1S_S4A showed relatively weak binding to the ORs compared with WT RTP1S (Fig. S3).

Altogether, these results suggested that the four N-terminal amino acids of RTP1S play an important role in the efficient traffic of ORs, although each of the residues differentially interacts with different ORs in trafficking.

### Dimerization of RTP1S_C2

**Figure 7.**
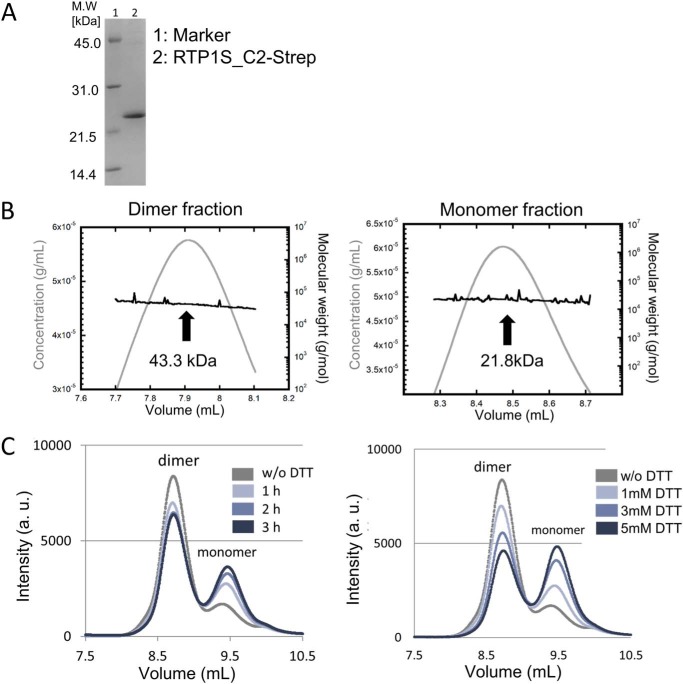
**Oligomeric state of RTP1S_C2-Strep.** *A,* SDS-PAGE image of purified RTP1S_C2-Strep. *B,* SEC-MALS analysis of the purified RTP1S_C2-Strep. *Left,* homodimer fraction, *Right,* monomer fraction. *C,* HPLC analysis with the addition of DTT. Reaction time dependence on 1 mm DTT (*left*) and DTT concentration dependence in the 1-h reaction (*right*) is shown.

To study the detailed mechanism of RTP1S, we tried to express and purify the TM domain deletion mutant RTP1S_C2. RTP1S_C2, with a Strep-tag at the C terminus (RTP1S_C2-Strep), was then expressed in *Escherichia coli*. RTP1S_C2-Strep was obtained in the soluble fraction and was purified by multiple chromatographies (Fig. 7*A*). In size-exclusion chromatography–multiangle laser light-scattering (SEC-MALS), the purified RTP1S_C2-Strep appeared as two peaks, the major one with a molecular mass of 43.3 kDa and the minor one with a molecular mass of 21.8 kDa (Fig. 7*B*). Given that the estimated molecular mass of RTP1S_C2-Strep was 22.0 kDa, these are likely to correspond to dimer and monomer forms, respectively. We also expressed and purified another TM deletion mutant, RTP1S_C3, with a Strep-tag at the C terminus (RTP1S_C3-Strep). The recombinant RTP1S_C3-Strep seemed to form larger oligomers (Fig. S4).

Incubation with dithiothreitol (DTT) induced a decrease in the dimer with an increase in the monomer (Fig. 7*C*). The effect changed with the incubation time and the concentration of DTT. It is plausible that the dimer formation of RTP1S_C2-Strep is mediated by disulfide bonds. The peak for the oligomeric RTP1S_C3-Strep also decreased with the addition of DTT, and the elution time of that peak was delayed with the increase in DTT concentration (Fig. S4*C*). Thus, disulfide bonds are also responsible for the multimerization of RTP1S_C3-Strep.

### Cys-2 is the dimerization site located at the intercellular domain of RTP1S

**Figure 8.**
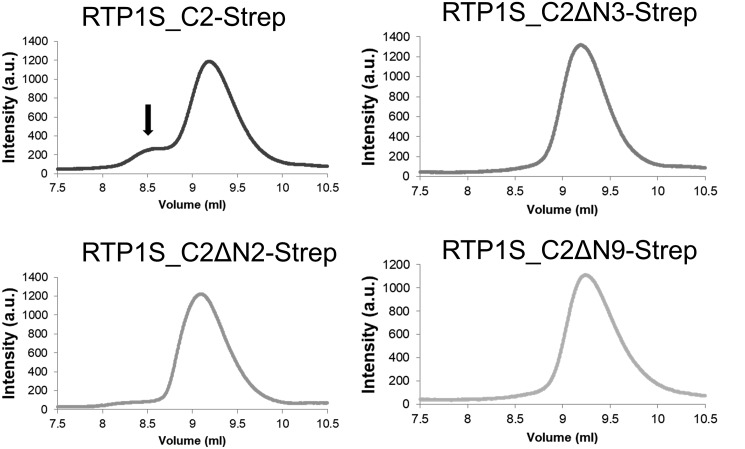
**Effect of N-terminal truncations on the dimer formation of RTP1S_C2-Strep.** Size-exclusion chromatograms of RTP1S_C2-Strep N-terminal truncation mutants are shown. The *black arrow* indicates the dimer form of RTP1S.

We also expressed and purified RTP1S_C2-Strep mutants with the truncation of the N-terminal amino acids. Size-exclusion chromatography analyses on HPLC showed that the dimer formation of RTP1S_C2-Strep disappeared only after deleting the 2nd amino acid, Cys-2 (RTP1S_C2ΔN2-Strep) (Fig. 8). These results, together with the results that the addition of DTT reduced the dimers (Fig. 7*C*), suggested that Cys-2 plays a critical role in the homodimerization of RTP1S.

FACS analysis showed that the cell-surface expression levels of three ORs by the RTP1S mutant that lacks Cys-2 (RTP1SΔN2) decreased by 54–67% (Fig. 2), and the RTP1S_C2A mutant also decreased by 40–84% (Fig. 4), suggesting that efficient trafficking requires Cys-2.

### Homotypic interaction of RTP1S in living cells

**Figure 9.**
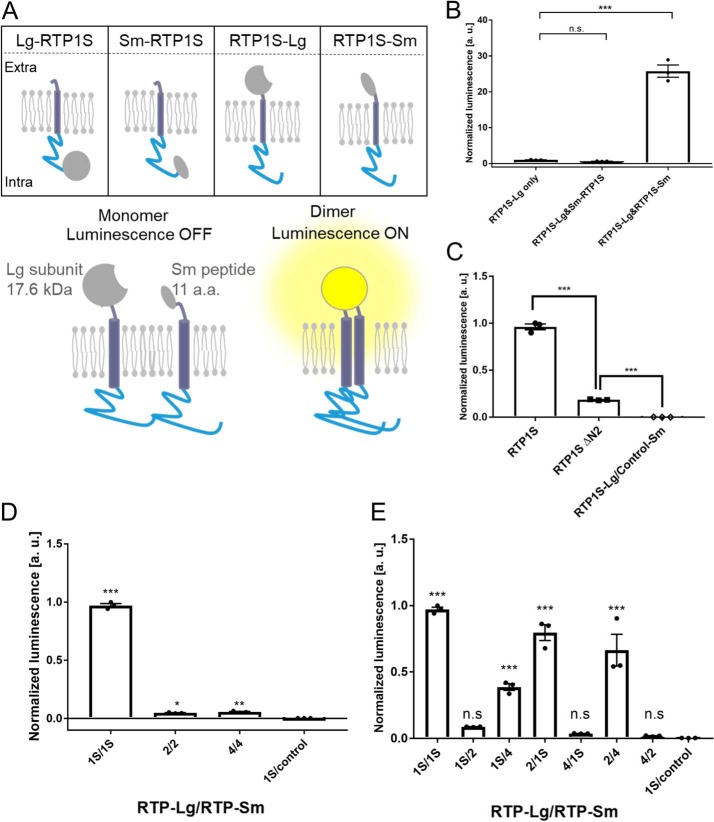
**Dimerization of RTP1S in living cells.** *A,* schematic diagram of the Nanobit assay against RTP1S in HEK293T cells. *a.a.*, amino acid. *B,* optimization of Nanobit against RTP1S fused to each Nanobit protein. Values of RTP1S-Lg only were used as the standard for multiple comparisons. *C,* Nanobit assay using RTP1SΔN2 fused with the Nanobit proteins at the C terminus. *D,* homodimerization of RTP2 and RTP4. Nanobit proteins were fused on the C terminus. *1S/1S* indicates the coexpression of RTP1S-Lg and RTP1S-Sm. *E,* heterodimerization of RTP proteins. Nanobit proteins were fused to the C terminus of the RTPs. *1S/1S* indicates the coexpression of RTP1S-Lg and RTP1S-Sm. Values of RTP1S-Lg only were used as the standard for multiple comparisons. The *error bar* indicates S.E.M. (*n* = 3). Multiple comparisons were performed using one-way analysis of variance followed by Tukey's (for *B* and *C*) and Dunnett's (for *D* and *E*) multiple comparison test (*, *p* < 0.05; **, *p* < 0.01; ***, *p* < 0.001; *n.s.*, not significant).

We examined RTP1S dimer formation in mammalian cells by a binary split-luciferase complementation method using NanoLuc Technology (23). In this method, split fragments of NanoLuc luciferase, a larger protein domain (Lg) and a small peptide (Sm), are fused with proteins whose interaction is subjected to study. We constructed RTP1S fused with Lg or Sm at the N or C termini (Fig. 9*A*). The protein expression levels of all four RTP1S variants were almost the same as that of RTP1S when they were expressed by the pCI vector (Fig. S5*A*). Among them, only RTP1S fused with Lg at the N terminus (Lg-RTP1S) had lost OR trafficking ability (Fig. S5*B*).

The RTP1S fusion variant genes were inserted into the pBit vector to make the expression level suitable for the assay. All combinations of RTP1S fusion variants were coexpressed in HEK293T cells. Among them, significantly high bioluminescence associated with complementation of split Nanoluc was observed when RTP1S-Lg and RTP1S-Sm were coexpressed (Fig. 9*B*). However, complementation of Nanoluc was not observed in the combinations of the RTP1S variants across the membrane (Sm-RTP1S/RTP1S-Lg) (Fig. 9*B*). These results indicated that RTP1S proteins interact with one another, consistent with the idea that it forms a homodimer in mammalian cells.

Because the purified RTP1S_C2ΔN2 did not form the homodimer (Fig. 7*B*), we examined whether RTP1SΔN2 shows a homotypic interaction. The bioluminescence of the cells expressing RTP1SΔN2 fused with Lg and Sm at the C terminus was significantly lower than that of RTP1S (Fig. 9*C*). However, RTP1SΔN2 still exhibited bioluminescence, which means that it has a homotypic interaction at the C terminus. The FKBP–Sm fusion protein as a control did not show a nonspecific interaction with RTP1S–Lg (1S/control in Fig. 9*C*). From these results, it was presumed that there are other parts involved in the dimer formation of RTP1S. The 2nd Cys residue located in the cytosolic domain seemed to be required for efficient dimerization of RTP1S.

### Heterotypic interaction of other RTP family proteins

Next, we examined whether other RTP family proteins, RTP2 and RTP4, also show homotypic interactions. RTP2 or RTP4 fused with Lg and Sm at their C termini were coexpressed, and the bioluminescence was measured (Fig. 9*D*). The results showed homotypic interactions of RTP2 and RTP4, although the efficiencies seem to be lower than that of RTP1S. Then we examined whether RTP proteins can form heteromers. Our data show that RTP proteins form heteromers with each other (Fig. 9*E*). These results suggested that homo- and heterotypic dimerization of RTP is a property common to RTP family proteins.

In the olfactory sensory neuron cells, RTP1S and RTP2 are expressed and transport ORs into the plasma membrane (10). Therefore, it is probable that not only the homodimers of RTP1S and RTP2 but also the heterodimers are present in the OSNs.

### Interspecies conserved amino acids at the N-terminal region of RTP1S

In the eight N-terminal amino acids of RTP1S, six residues, including Cys-2, are conserved between various mammalian species (Fig. S6), supporting the idea that Cys-2 plays a conserved role in RTP1S function in mammals.

**Figure 10.**
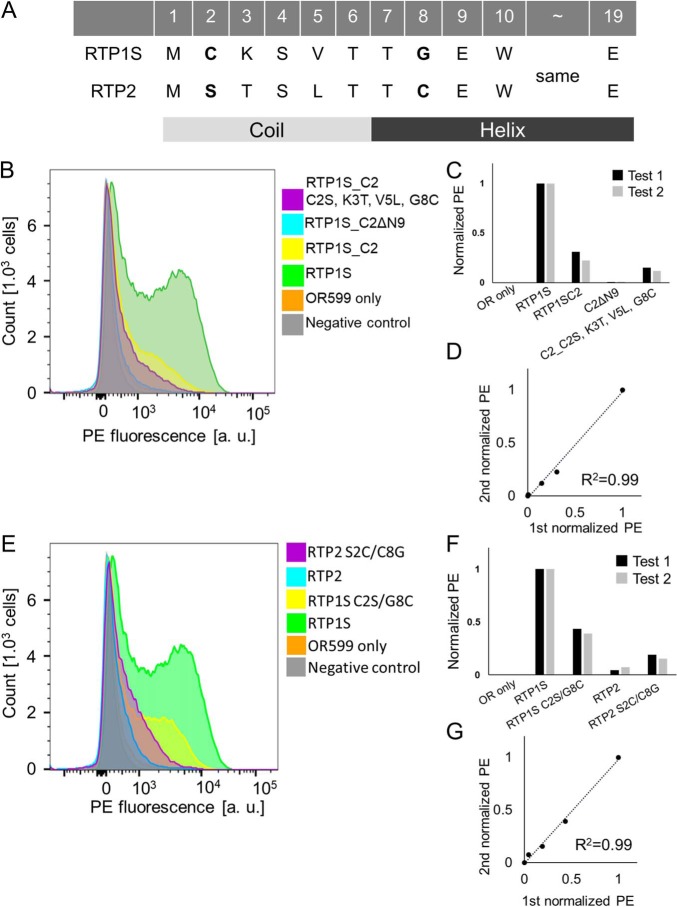
**Importance of conserved amino acids at the N-terminal region of RTP1S.** *A,* amino acid sequence of the N termini of RTP1 and RTP2. Secondary structure was predicted with Jpred4 (37). FACS analyses are shown for the N-terminal deletion (ΔN9) and chimera (C2S, K3T, V5L, and G8C mutants) of RTP1S_C2 against Olfr599 (*B*) and cysteine replacement mutant of RTP1S and RTP2 against Olfr599 (*E*). Each combination of mutants of RTP1S and Olfr599 was transfected into HEK293T cells, and the cell-surface expression levels were measured. *x* axis, PE fluorescence; *y* axis, cell number. *C* and *F,* normalized geometric mean of PE fluorescence of each measurement was used for comparison. The values of RTP1S and OR only were used as the positive (= 1) and the negative controls (= 0), respectively. *D* and *G,* comparison of the normalized response by different experiments. *R*^2^ values were calculated by Pearson's correlation coefficient from the linear regression analysis.

RTP1S and RTP2 share 74% (163/221) identity and 81% (180/221) similarity in the mouse. The OR trafficking ability of RTP2 seems to be lower than that of RTP1S (10). Four amino acids, including Cys-2, were different between RTP1S and RTP2 in the eight N-terminal amino acids (Fig. 10*A*). Truncation of the four N-terminal amino acids of RTP1S_C2 (RTP1S_C2ΔN4) caused a loss in traffic activity for Olfr599 (Fig. 3) (21). The RTP1S mutant with the same N-terminal amino acid sequences as RTP2 (C2S, K3T, V5L, and G8C) exhibited a decreased ability (Fig. 10, *B–D*). The two amino acid replacements in RTP1S (C2S/G8C) significantly impaired OR-transporting activity (43.6% against RTP1S) (Fig. 10, *E–G*). In contrast, the RTP2 mutant with the two amino acid replacements (S2C/C8G) exhibited enhanced activity (157% against RTP2) (Fig. 10, *E–G*).

Together, these data suggest that Cys-2 and Ser-4 in the N terminus of RTP1S are especially important for efficient trafficking of ORs. However, the minimum elements of RTP1S required for OR transport into the cell membrane differ depending on the OR of the transport partner.

## Discussion

In this study, we conducted functional and structural analyses of RTP1S to unveil the mechanism of OR transport to the plasma membrane. We showed that RTP1S forms a homodimer and that specific residues located at the N terminus play essential roles in trafficking ORs.

This is the first report on the homodimer formation of RTP1S evidenced by both recombinant protein analysis and living cell assays. The 2nd cysteine deletion or replacement decreased the levels of RTP1S dimerization and decreased the level of ORs localized on the cell surface (Figs. 2, 4, and 8) but maintained OR-transporting activity. We suggest that the dimer formation of RTP1S is important for efficient OR trafficking but is unlikely to be indispensable. However, we cannot determine the oligomer stoichiometry of RTP1S in a living cell because the split luciferase assay works if the targeted protein forms any multimers. The stoichiometry of RTP1S and ORs in the cellular membrane has been unknown. It is known that many class A GPCRs form homodimers (24–30). Although the homo-oligomeric state of ORs has not yet been observed, ORs form a heterocomplex with a non-OR GPCR, the muscarinic M3 receptor (31). M3-R facilitates G-protein–mediated signal transduction induced by activated ORs (32). It is known that dimers and oligomers of ORs are detected by SDS-electrophoresis using extracts from cells expressing ORs (33). The homo-oligomeric state of RTP1S may be linked to promoting the functional activity of ORs transported into the cell membrane. In light of our current study, it will be interesting to study whether ORs form dimers or even larger multimers through their interactions with RTP dimers and M3-R.

A previous study with RTP1 and RTP2 double-knockout mice showed that some ORs could independently localize and be functional in the cell membrane during OSN maturation without RTP1 and RTP2 (12, 34, 35). In this study, we showed that ORs exhibit different interactions with RTP1S for their trafficking in heterologous cells (Figs. 5 and 6). N-terminal truncation and the TM domain deletion mutants of RTP1S could transport Olfr599. In contrast, Olfr1484 failed to localize to the cell membrane for all TM domain deletion mutants (Figs. 1 and 5). All RTP1S mutants were more effective with Olfr1377 than other ORs in promoting cell-surface expression. Together, our data suggest that different ORs interact with RTP1S through different mechanisms. The difference in the amino acid sequence of each OR and the stability of their structure may cause the difference in the functionality of RTP1S. Future studies should address how individual residues of RTP1S interact with different ORs and how these interactions affect OR trafficking.

Evaluation of the functional activity of recombinant RTP1S is difficult because we used indirect methods such as OR trafficking or responsiveness. Although it is thought that ORs become structurally stable by interacting with RTP1S during membrane transport, the structure of RTP1S itself may also be stabilized by the interaction with ORs.

For further studies, high-resolution structural analyses (*i.e.* X-ray crystallography, NMR, or cryo-EM) of RTP proteins would allow us to conduct various approaches, including protein–protein docking and molecular dynamics simulation, for investigating the interaction between RTP and ORs using the 3D theoretical model of ORs. Further analysis is necessary to unveil the membrane transport mechanism of OR by RTP1S to understand how RTPs transport hundreds of ORs from the endoplamsic reticulum to the plasma membrane.

## Experimental procedures

### Expression and purification of the TM domain deletion mutants of RTP1S in E. coli

All DNAs for *Rtp1S* variants with the Strep-tag sequence (WSHPQFEK) at the C terminus were subcloned into the pET23b vector. They were expressed in *E. coli* BL21 star (DE3) cells. Purification of RTP1S_C2-Strep and RTP1S_C3-Strep was carried out by several steps of chromatography using StrepTactin–Sepharose high performance (GE Healthcare, Buckinghamshire, UK), Resource Q (GE Healthcare), and a gel-filtration column Superdex 75 (GE Healthcare). Purified RTP1S_C2-Strep and RTP1S_C3-Strep in 20 mm phosphate buffer, pH 8.0, with 100 mm NaCl were concentrated by ultrafiltration and used for analysis immediately.

### SEC-MALS

The purified RTP1S_C2-strep and RTP1S_C3-Strep were analyzed using an HPLC system with OH-Pak SB-804HQ (Showa Denko, Tokyo, Japan). An aliquot (100 μl) of the RTP1S solution (0.3 mg/ml) was injected onto the column with buffer (20 mm phosphate buffer, pH 8.0, 100 mm NaCl). Measurement of molecular mass was carried out under the same conditions by SEC-MALS using a multiangle light-scattering detector (mini-DAWN DSP, Wyatt Technology) and a differential refractive index detector (Shodex RI-71, Showa Denko).

### Cell culture

HEK293T cells purchased from Thermo Fisher Scientific (Waltham, MA) were maintained in minimum essential medium Eagle's (Sigma-Aldrich Japan, Tokyo, Japan) containing 10% fetal bovine serum (HyClone, GE Healthcare) and 0.5% antibiotic/antimycotic mixed stock solution (Nakalai Tesque, Kyoto, Japan) at 37 °C in 5% CO_2_.

### Split luciferase reconstruction assay

We used the Nano-Glo Live Cell Assay System (Promega. Madison, WI) for the split luciferase reconstruction assay. HEK293T cells were plated on poly-d-lysine–coated 96-well plates (Thermo Fisher Scientific). Plasmid DNAs were transfected using Lipofectamine 2000 (Invitrogen). Then, each *Rtp1S* was subcloned into the pBiT1.1-C (TK/LgBiT), pBiT2.1-C (TK/SmBiT), pBiT1.1-N (TK/LgBiT), and pBiT2.1-N (TK/SmBiT) vectors. Combinations of plasmids for RTP1S fusion variants were transfected into HEK293T cells. After 24 h post-transfection, the medium was replaced with 40 μl of 10 mm HEPES, and we followed the manufacturer's protocols for measuring luciferase activities. Luminescence was measured using a GloMax-Multi Detection System (Promega).

### Plasmid construction for expression of ORs and RTP1S mutants in HEK293T cells

The DNA fragment for the Rho-tag (MNGTEGPNFYVPFSNATGVVR) was subcloned into the pCI mammalian expression vector (Promega). Then, the genes *Olfr1484*, *Olfr1377*, and *Olfr599* were subcloned downstream of the Rho-tag DNA fragment. The *Rtp1S* gene was inserted into the pCI expression vector without the Rho-tag (pCI-RTP1S). Through PCR-based mutagenesis on pCI-RTP1S, plasmids for RTP1S mutants were constructed. The sequences of all plasmids were verified by sequencing.

### Luciferase assay and data analysis

The Dual-Glo luciferase assay system (Promega) was used for the luciferase assay (15). HEK293T cells were plated on poly-d-lysine–coated 96-well plates (Thermo Fisher Scientific). Plasmid DNAs of ORs and RTP1S were transfected using Lipofectamine 2000 (Invitrogen). In addition, two luciferase constructs were used, including a firefly luciferase gene driven by a cAMP-response element (CRE-Luc) and a *Renilla* luciferase gene driven by a constitutively active SV40 promoter (pRL-SV40) that was used as an internal control for cell viability and transfection efficiency. For each 96-well plate, 10 ng of CRE-Luc, 5 ng of pRL-SV40, 5 ng of OR, and 5 ng of RTP1S were transfected. Twenty four hours post-transfection, the medium was replaced with 25 μl of odorant solution diluted in CD293 (Invitrogen) and incubated for 3.5 h at 37 °C in 5% CO_2_. We followed the manufacturer's protocols for measuring firefly luciferase (Luc) and *Renilla* luciferase (RL) activities. Luminescence was measured using a GloMax-Multidetection System (Promega). The relative response of ORs was calculated as (Luc/RL − Luc/RL_min_)/(Luc/RL_max_ − Luc/RL_min_), where Luc/RLN represents the mean value from the replicate wells of a certain sample, and Luc/RLmin represents the mean value from the minimal response in the experiment.

### FACS analysis

HEK293T cells were seeded in 35-mm dishes and then transfected with 1200 ng of an OR expression plasmid and 300 ng of an RTP expression plasmid per dish. A total of 30 ng of a GFP expression plasmid was also transfected per dish as a control for transfection efficiency. At 24 h post-transfection, the cells were dissociated in Cellstripper (Corning, Corning, NY) and washed with staining buffer (PBS containing 2% (v/v) fetal bovine serum and 15 mm NaN_3_). Then, the cells were transferred to a tube for incubation with the anti-rhodopsin 4D2 antibody (Merck Millipore, Billerica, MA) for 45 min on ice and then with phycoerythrin (PE)-conjugated donkey anti-mouse IgG (Jackson ImmunoResearch Laboratories, West Grove, PA) for 30 min on ice. Each antibody was diluted in staining buffer. To stain dead cells, 7-amino-actinomycin D (Merck Millipore) was added before analysis. The cells were analyzed using a BD FACS Canto II flow cytometer (BD Biosciences, San Jose, CA) and a BD LSRFortessa cell analyzer (BD Biosciences) with gating allowing for 10,000 GFP-positive, single, spherical, and viable cells, and the measured PE intensities were analyzed and visualized using FlowJo (15, 36).

### Immunoprecipitation

HEK293T cells in 35-mm dishes were transfected with the plasmids to express the N-terminally Rho-tagged OR and RTP1S variants and/or the negative control. Twenty four hours after transfection, cells were lysed with RIPA buffer (50 mm Tris, pH 8.0, 150 mm NaCl, 1% Nonidet P-40, 0.5% sodium deoxycholate, and 1× complete protease inhibitor mixture (Merck Millipore)). The lysates were incubated with Dynabeads (Thermo Fisher Scientific) conjugated with the anti-Rho4D2 antibody (Merck Millipore) for 10 min at room temperature and washed with PBS buffer, pH 7.4, containing 0.02% Tween 20. The bound proteins were eluted by incubation with 1× SDS sample buffer at room temperature for 2 h and at −80 °C overnight and then subjected to Western blotting.

### Western blotting

Proteins from the whole-cell lysates or immunoprecipitants were resolved by SDS-PAGE and subsequently electrophoretically transferred onto polyvinylidene difluoride membranes using the trans-blot turbo transfer system (Bio-Rad). The membranes were blocked and incubated with primary antibodies (anti-Rho4D2 antibody or anti-RTP1 antibody (Invitrogen)) followed by horseradish peroxidase-conjugated secondary antibodies (MBL Life Science, Nagoya, Japan) using iBind Western Systems (Thermo Fisher Scientific). The signals were detected using Western BLoT Quant HRP substrate (Takara Bio, Shiga, Japan) using the C-DiGit System (LI-COR Biosciences, Lincoln, NE) according to the manufacturer's instructions.

### Amino acid sequence analysis

Amino acid sequences of RTP1S from various animals were chosen from the NCBI database. The animal species and accession numbers we used were as follows: mouse, ABU23737.1; human, AAT70680.1; rat, NP_001099340.1; dog, XP_025307127.1; gorilla, XP_004038226.1; flying fox, XP_006925887.1; Chinese hamster, XP_007606938.1; macaque, NP_001270896.1; horse, XP_001498142.1; cow, NP_001179185.1; star-nosed mole, XP_004675139.1; opossum, XP_001366241.1; koala, XP_020844727.1; and platypus, XP_001512958.1. The phylogenic tree and the amino acid alignment were analyzed using the ClustalW program.

### Statistical analysis

Multiple comparisons were performed using one-way or two-way analysis of variance using GraphPad Prism (GraphPad Software, San Diego). Student's *t* test was performed using the built-in function in Microsoft Excel. The average is shown as the mean ± S.E.

## Author contributions

Y. F. and M. Y. conceptualization; Y. F., R. T., R. I., T. K., S. S., and K. I. data curation; Y. F. and R. T. formal analysis; Y. F., H. M., and M. Y. supervision; Y. F. and M. Y. funding acquisition; Y. F., R. T., R. I., and K. I. investigation; Y. F., I. O., H. M., and M. Y. methodology; Y. F. writing-original draft; Y. F. and M. Y. project administration; Y. F., H. M., and M. Y. writing-review and editing; R. T. validation; I. O. resources.

## Supplementary Material

Supporting Information

## Footnotes

4 The abbreviations used are:

OR: odorant receptor
OSN: olfactory sensory neuron
GPCR: G-protein–coupled receptor
SEC-MALS: size-exclusion chromatography multiangle laser light scattering
PE: phycoerythrin
RTP: receptor-transporting protein
TM: transmembrane
Lg: larger protein domain
Sm: small peptide
Luc: luciferase
RL: *Renilla* luciferase
M3-R: M3 receptor.
