# Supplementary material for: The N-terminal region of RTP1S plays important roles in dimer formation and odorant receptor-trafficking
Source: J Biol Chem. 2019 Aug 8;294(40):14661–73. doi: 10.1074/jbc.RA118.007110 (PMC6779431; doi:10.1074/jbc.RA118.007110)
Supplement: Supporting Information [file supp_294_40_14661__index.html]

The N-terminal region of RTP1S plays important roles in dimer formation and odorant receptor-trafficking — Role of N-termini of RTP1S in its dimer form and OR-traffic — The N-terminal region of RTP1S plays important roles in dimer formation and odorant receptor-trafficking — Role of N terminus of RTP1S in its dimer form and OR traffic — Supporting Information 

# The N-terminal region of RTP1S plays important roles in dimer formation and odorant receptor-trafficking

## Supporting Information

- Supporting Information (to be published online) - Supplementary Figures
