# Supplementary material for: The N-terminal region of RTP1S plays important roles in dimer formation and odorant receptor-trafficking
Source: J Biol Chem. 2019 Aug 8;294(40):14661–73. doi: 10.1074/jbc.RA118.007110 (PMC6779431; doi:10.1074/jbc.RA118.007110)
Supplement: Supporting Information [file supp_RA118.007110_142492_2_supp_368483_pvftz9.pdf]

Supporting Information

The N-terminal region of RTP1S plays important roles in dimer formation and odorant receptor-trafficking

Yosuke Fukutani, Ryohei Tamaki, Ryosuke Inoue, Tomoyo Koshizawa, Shuto Sakashita, Kentaro Ikegami, Ikuroh Ohsawa, Hiroaki Matsunami, Masafumi Yohda

Contents

Supplementary Fig. S1

Surface expression of the Rho-tagged ORs cotransfected with the N-terminal truncation mutants of RTP1S_C3

Supplementary Fig. S2

Protein expression of RTP1S mutants in HEK293T cells.

Supplementary Fig. S3

Interactions of RTP1S mutants with Olfr599.

Supplementary Fig. S4

Oligomeric state of RTP1S_C3-Strep

Supplementary Fig. S5

Effect of the fusion with a split luciferase fragment on the expression and the function of RTP1S

Supplementary Fig. S6

Comparison of amino acid sequences of RTP1Ss

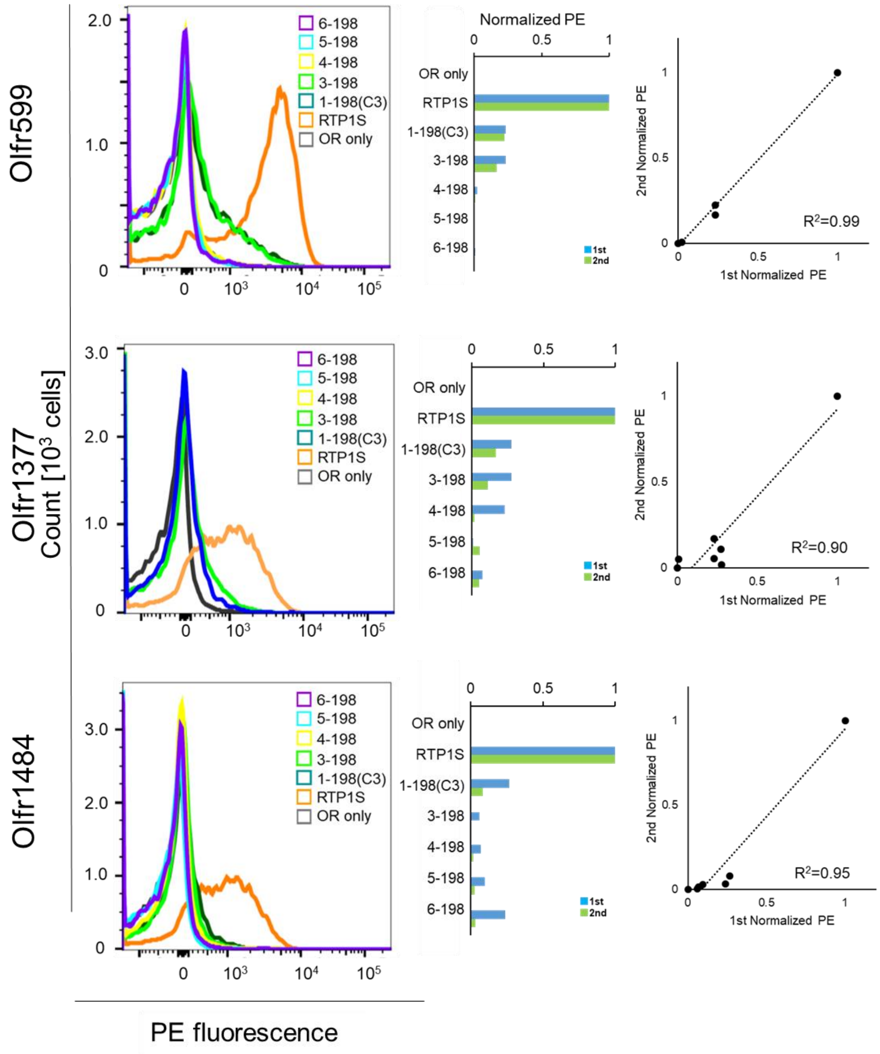

Supplementary Fig. S1 Surface expression of the Rho-tagged ORs cotransfected with the N-terminal truncation mutants of RTP1S_C3

Each combination of mutant RTP1_C3s and ORs (Olfr599, Olfr1377 and Olfr1484) was transfected into HEK293T cells and the cell surface expression level was measured. Results from two independent experiments were shown in each graph. X-axis: the PE fluorescence, Y-axis: cell number. Geometric mean of PE fluorescence of each measurement were used for comparison. The values of RTP1S and OR only were used as positive (=1) and negative control (=0), respectively. Comparison of the normalized response by different experiments. R2 values were calculated by Pearson's correlation coefficient from the linear regression analysis.

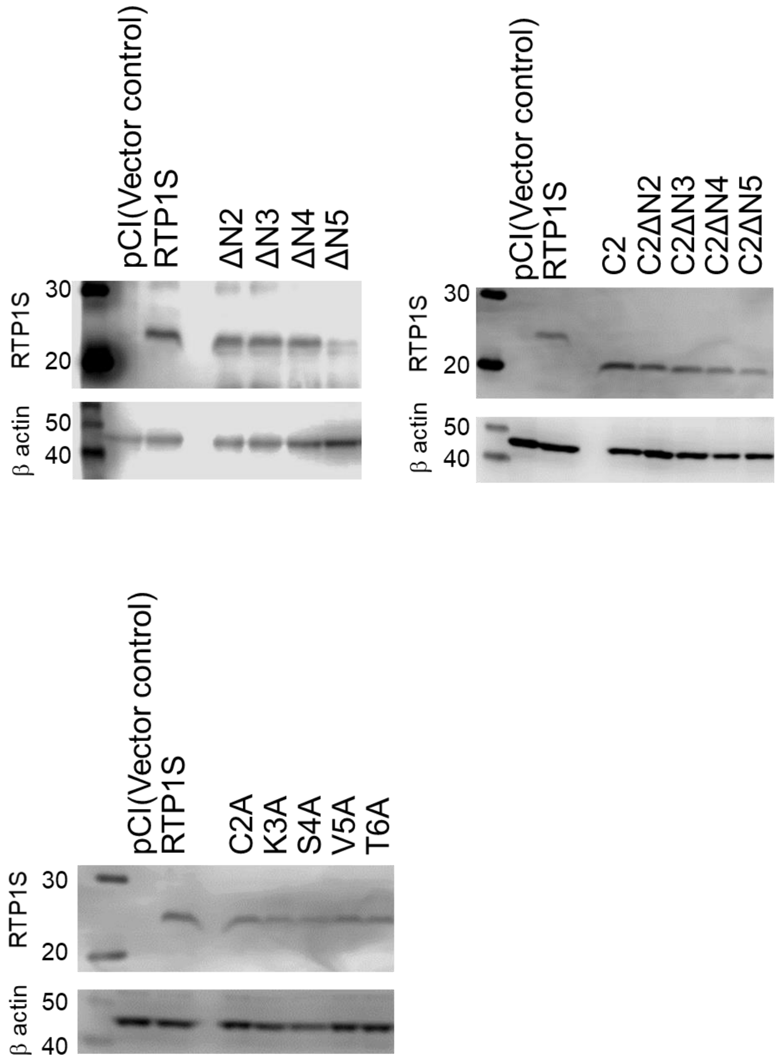

Supplementary Fig. S2 Protein expression of RTP1S mutants in HEK293T cells.

Whole cell lysates of HEK293T cells transfected with RTP1S mutants were analyzed by Western blotting using anti-RTP1 or beta actin antibody.

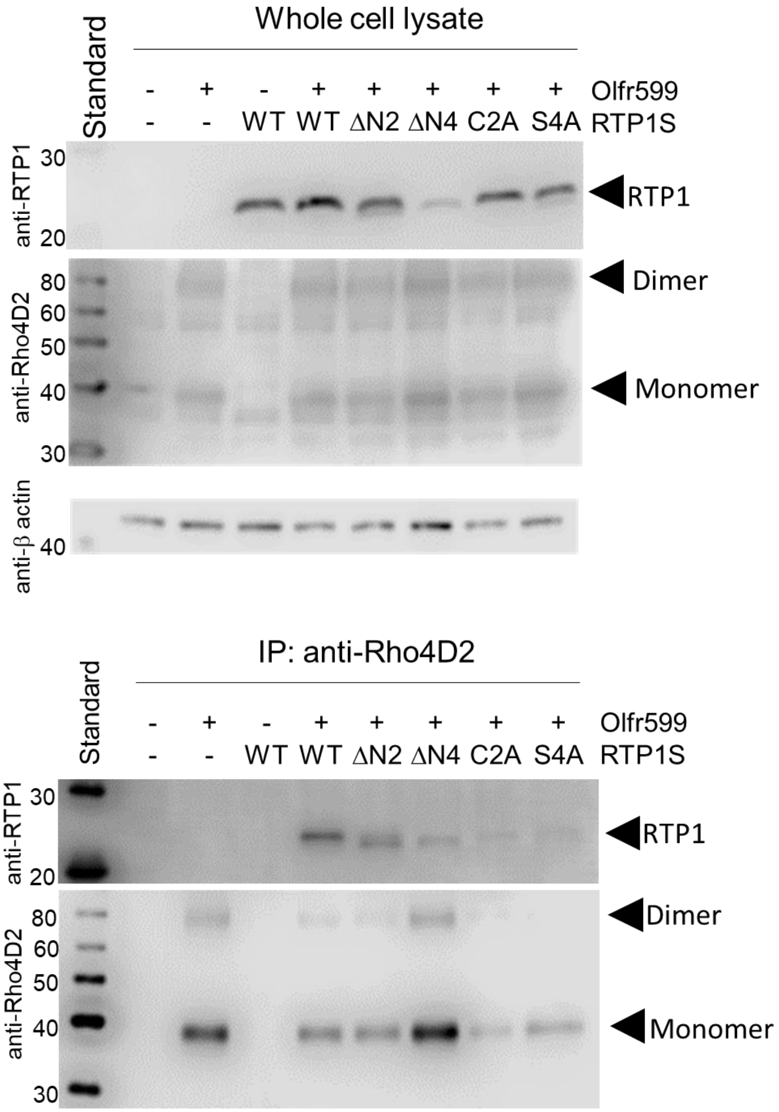

Supplementary Fig. S3 Interactions of RTP1S mutants with Olfr599.

Whole cell lysates of HEK293T cells transfected with Rho-tagged Olfr599 and/or each RTP1S mutant (Upper) or their immunoprecipitants with anti-Rho4D2 antibody (Bottom) were analyzed by Western blotting using anti-Rho4D2, anti-RTP1 or beta actin antibody.

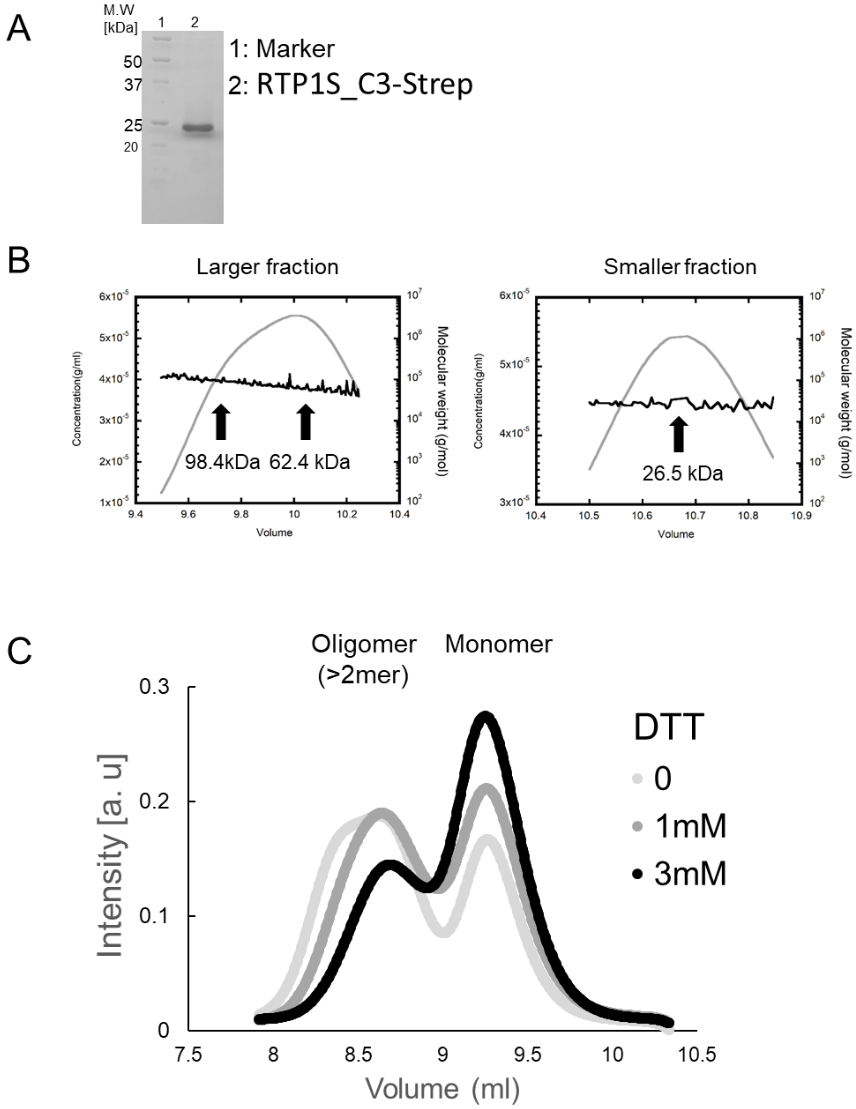

Supplementary Fig. S4 Oligomeric state of RTP1S_C3-Strep

(A) SDS-PAGE image of the purified RTP1S_C3Strep. (B) SEC-MALS analysis of RTP1S_C3-Strep. Left: Larger fraction, Right: Smaller fraction. (C) The effect of DTT. Chromatograms at after 1 hr from the addition of DTT are shown.

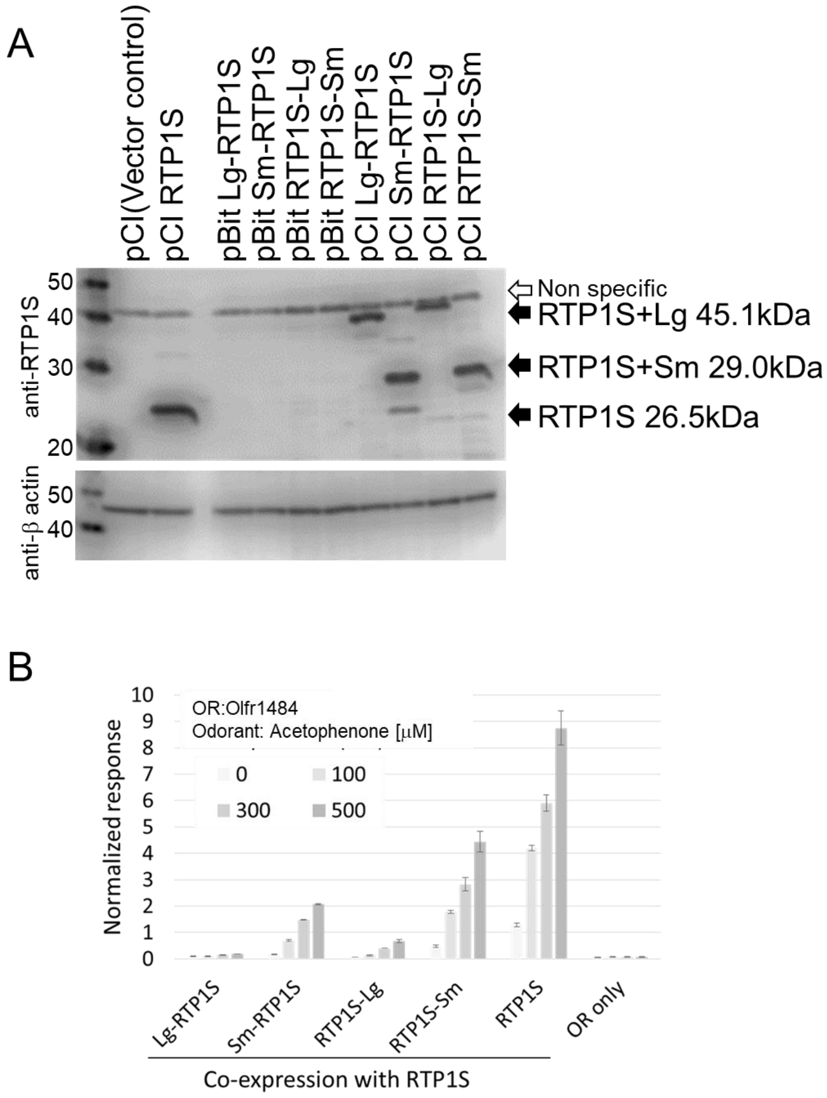

Supplementary Fig. S5 Effect of the fusion with a split luciferase fragment on the expression and the function of RTP1S

(A)Expression of RTP1S fused with split luciferase

Whole cell lysate of HEK293T cells transfected with RTP1S fused with Lg or Sm were blotted with anti-RTP1 or beta actin antibody.

(B)Ligand response of Olfr1484 with the co-expression of RTP1S fused with Lg or Sm, as indicated by cAMP-mediated luciferase assays. Acetophenone was used the odorant as one of the known agonist of Olfr1484.

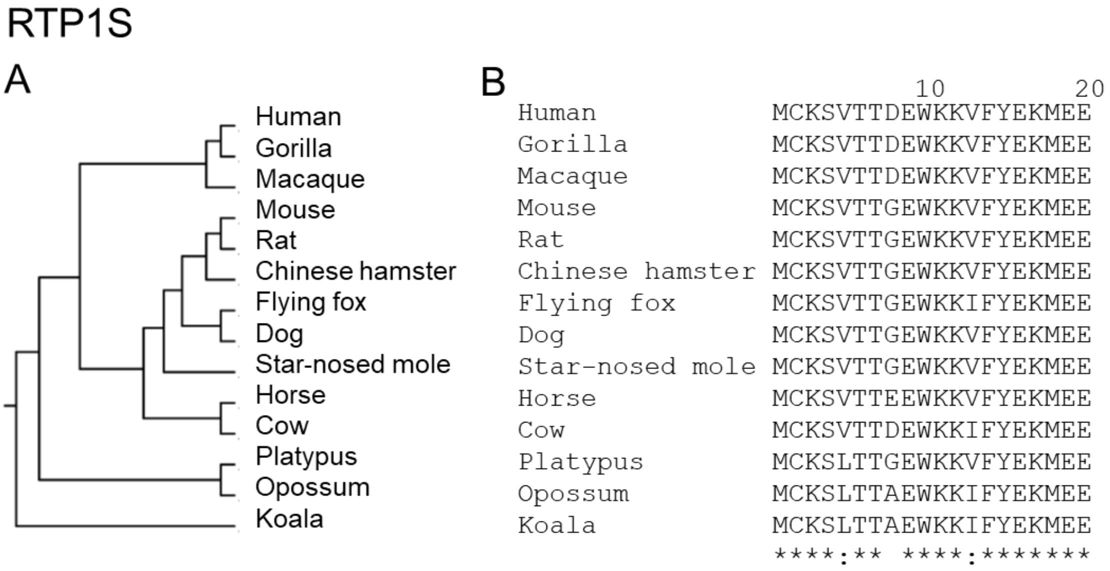

Supplementary Fig. S6 Comparison of amino acid sequences of RTP1Ss

Amino acid sequences of RTP1Ss from 14 vertebrates were compared by ClustalW Phylogenetic tree analysis (A) and amino acid sequence alignment in the N-terminus (B) are shown.
